# Validity of the Tree Drawing Test Suicide Ideation Assessment Scale in adolescents

**DOI:** 10.3389/fpsyt.2025.1701452

**Published:** 2025-12-09

**Authors:** Yanfei Zhang, Yige Liu, Wei Liu, Lvping Sun, Feng Leng, Ziyang Li, Guorui Liu

**Affiliations:** 1 Department of Medical Psychology, Second Affiliated Hospital of Naval Medical University, Shanghai, China; 2 Institute of Applied Psychology, Jiangsu University, Zhenjiang, China; 3 Medical Affairs Office, Second Affiliated Hospital of Naval Medical University, Shanghai, China; 4 School of Health and Nursing, Zhenjiang College, Zhenjiang, China; 5 Department of Medical Psychology, Hospital of PLA Navy, Shanghai, China; 6 Sleep Medicine Center, Suzhou Guangji Hospital, Suzhou, China

**Keywords:** projective test, Tree Drawing Test, Suicide Ideation Assessment, adolescents, reliability and validity

## Abstract

**Objective:**

To examine the validity of the Tree Drawing Test Suicide Ideation Assessment Scale (TDT-SIAS) for screening suicidal ideation in adolescents.

**Methods:**

A case–control design was employed, involving 36 adolescents with suicidal ideation and 58 adolescents without suicidal ideation. All participants completed the Tree Drawing Test, and their responses were analyzed using computer-based image recognition and data acquisition techniques with the TDT-SIAS. Statistical analyses were then conducted.

**Results:**

Significant differences were observed in total scores between the suicidal ideation group and the non-suicidal group (p < 0.001). Using a cutoff score of 9, the Youden Index was 0.901, with a sensitivity of 0.935, specificity of 0.966, positive predictive value of 0.956, and negative predictive value of 0.949. Inter-rater reliability (Cohen’s kappa) ranged from 0.779 to 0.961.

**Conclusion:**

The TDT-SIAS demonstrated good screening performance and effectively distinguished adolescents with suicidal ideation from those without. It may serve as a preliminary screening tool for adolescent suicidal ideation. However, limitations of this study include the relatively small sample size and the lack of consideration for cultural differences, which may affect generalizability. Future studies should expand sample size and examine cross-cultural applicability.

## Introduction

Suicide is both a major health problem and a pressing social issue, causing profound losses to individuals, families, and society. Among adolescents, suicidal behaviors constitute an especially critical global public health concern. Epidemiological studies have shown that suicide is one of the leading causes of death among adolescents, and suicidal ideation is a strong predictor of both suicidal behavior and suicide mortality (1, 2). Therefore, early identification of high-risk adolescents and timely intervention are essential for suicide prevention. However, suicidal thoughts and behaviors in adolescents are often concealed. Although existing screening tools have demonstrated some utility, they rely heavily on self-report, making them vulnerable to response bias (3). This highlights the urgent need to develop supplementary assessment methods that are both accessible and suitable for adolescents.

Currently, methods for assessing adolescent suicidal ideation primarily include self-report questionnaires, parent or teacher reports, and clinical interviews. Self-report instruments, such as the Suicidal Ideation Questionnaire (SIQ) and the Beck Scale for Suicidal Ideation (BSSI), allow for rapid quantification of suicide risk but are limited by their overt testing purpose. They are prone to social desirability bias, self-protective responses, and even intentional misreporting for secondary gain. Parent or teacher reports are constrained by the observer’s perspective and may fail to capture the adolescent’s private thoughts (4). Clinical interviews, while capable of yielding more in-depth information, are time-consuming, require a high level of professional expertise, and are impractical for large-scale screening.

Drawing-based projective tests analyze symbolic and structural features to explore personality traits and unconscious processes (5). As nonverbal, non-confrontational methods, they allow adolescents to express implicit psychological content naturally. With a long history of application, drawing projection tests have been widely employed in psychological assessment. Originally developed within the psychodynamic tradition, these tests were designed to explore an individual’s personality structure, emotional conflicts, and unconscious processes by analyzing the symbolic content and formal features of drawings. Although their initial purpose was to understand personality and emotional functioning, later research has shown that certain drawing features may also reflect depressive affect and suicidal ideation. Early studies suggested that they are valuable for evaluating unconscious material, emotional states, and psychological stress. Compared with traditional interviews and questionnaires, drawing tests can reflect an individual’s inner world through the creative process, making them particularly suitable for adolescents, especially when they have not yet fully developed verbal expression skills or are reluctant to express their emotions.

Among these, the Tree Drawing Test (TDT) has been extensively studied in clinical practice. In this test, participants are asked to draw a tree, and their psychological state is inferred through the analysis of features such as form, proportions, and details. Previous studies have demonstrated the utility of the TDT as an auxiliary diagnostic tool for schizophrenia, mania, depressive disorders, anxiety disorders, personality disorders, and Alzheimer’s disease (6–11). From a psychodynamic and projective perspective, different parts of the tree are viewed as symbolic representations of distinct psychological domains: the crown reflects the individual’s mental world and cognitive functioning; the trunk represents one’s emotional experience and developmental history; and the roots correspond to instinctual drives and unconscious foundations. When suicidal or depressive tendencies emerge, drawings may display weakened or distorted structural integration—such as fragmented trunks, shallow or absent roots, or disproportionately small crowns—symbolically reflecting emotional instability, psychological constriction, or disrupted connections between conscious and unconscious aspects of the self. Regarding its relationship with suicidal ideation, researchers have used the TDT to assess suicide risk in prison populations, finding that certain features—such as single-line drawings or trunks disproportionately larger than roots—were associated with suicidal behavior (12). These graphic features may represent a more severe form of psychological constriction and ego fragility: a single-line drawing suggests emotional withdrawal and reduced expressive energy, whereas an enlarged trunk with minimal roots reflects instability of the self and lack of psychological grounding, corresponding to the inner experiences of emptiness and hopelessness often observed in individuals with suicidal ideation. Other studies have reported that tree characteristics, including trunk size, root morphology, or branch structure, may be linked to emotional distress, depressive states, and even suicidal tendencies. These findings are consistent with the symbolic interpretations discussed above, suggesting that structural distortions in tree drawings may mirror psychological imbalance and emotional depletion commonly associated with depressive and suicidal states. These findings provide preliminary evidence for the potential of the TDT in assessing suicidal ideation. Nevertheless, despite this theoretical support, most studies to date have focused on analyzing isolated features, with limited efforts toward systematically integrating and standardizing indicators. Furthermore, research examining the validity of TDT-based suicidal ideation assessment in adolescent populations remains scarce.

In recent years, researchers have sought to develop structured scoring systems and quantitative methods to interpret projective drawings, thereby enhancing their reliability and validity in both clinical and research settings. Building on previous studies that examined the relationship between the Tree Drawing Test and depressive symptoms (13, 14), our research team proposed integrating multiple qualitative indicators related to depressive symptoms and suicidal ideation with standardized quantitative indices (e.g., the area and length of tree components). Based on this approach, we developed the Tree Drawing Test Suicide Ideation Assessment Scale (TDT-SIAS), designed to serve as a novel supplementary tool for identifying individuals at potential high risk. The primary objective of the present study was to evaluate the effectiveness of the TDT-SIAS in screening suicidal ideation among adolescents, with the ultimate aim of supporting early detection in this vulnerable population.

## Methods

This study was conducted at the Suzhou Mental Health Center in Suzhou, China. Participants included a clinical sample of adolescents with depressive disorders recruited from inpatient wards, and a control sample of healthy adolescents recruited from the community. Both groups were evaluated using the suicidality module of the Mini-International Neuropsychiatric Interview (M.I.N.I.), and subsequently classified into a suicidal ideation group and a non-suicidal ideation group. Recruitment took place between February 2021 and June 2022.

The inclusion of hospitalized adolescents with depressive disorders was intended to ensure the clinical validity of the instrument by capturing a population with a higher likelihood of suicidal ideation. However, we acknowledge that recruiting from an inpatient setting may introduce selection bias due to more severe symptom profiles or treatment exposure. To mitigate this, the control group was matched for key demographic variables (age, gender, and education) and recruited during the same period from the same geographical area.

### Ethics

The study was reviewed and approved by the Ethics Committee of Suzhou Guangji Hospital (Approval No. 2021-012). All participants and their legal guardians were fully informed of the study’s objectives and procedures prior to enrollment and provided written informed consent. Given the inclusion of adolescents with depressive disorders and potential suicidal ideation, a structured safety monitoring protocol was implemented throughout data collection. Participants identified as having suicidal ideation, as assessed by the M.I.N.I. suicidality module or through clinical interview, were immediately evaluated by a qualified psychiatrist. Appropriate crisis intervention measures were taken when necessary, including safety planning, enhanced observation, and communication with guardians. Participants requiring further intervention received timely referral to specialized mental health services within the hospital to ensure continuous clinical care and safety.

### Recruitment criteria

The depressive disorder group met the DSM-5 diagnostic criteria for major depressive disorder, with participants aged 12–17 years, regardless of sex, and a Hamilton Depression Rating Scale-21 (HDRS-21) score ≥ 21 (15). The healthy control group was recruited from the same geographic region and during the same time period (2021–2022), and was matched to the depressive disorder group by sex and age distribution. Inclusion criteria for controls were: no depressive symptoms based on DSM-5 diagnosis, no significant psychiatric symptoms (no positive factors on the Symptom Checklist-90, SCL-90), no history of psychiatric disorders.

### Measures

Tree Drawing Test: Each participant was provided with A4 paper and a black or dark blue pen. The following instructions were given:

1. The projective drawing test is not a test of drawing skills, and the quality of the drawing is not important.
2. The test is not a life drawing; the picture does not need to match real-world objects.
3. If the participant cannot draw what they want, they may draw a circle and write the Chinese characters for it.
4. Before drawing the tree, close your eyes and meditate for half a minute. Draw the tree that appears during meditation. If no tree appears, open your eyes and draw the tree you most want to draw.
5. After completing the drawing, write your age and gender on the paper (13).(Due to cultural differences in the assessment and interpretation of the Tree Drawing Test, this study is limited to participants from mainland China. The participants’ cultural background may influence the form and symbolic meaning of the tree depicted, and therefore, the potential impact of cultural background on the drawing content should be considered when interpreting the results (16)).

### Instruments

High-resolution scanner (Epson DS-1630): All tree drawings were scanned and digitized using an Epson DS-1630 scanner.

Tree Drawing Test Projection Analysis Software: A custom-developed software system, created by the research team, was used to automatically calculate and extract quantitative indices such as the length, width, height, and area of the crown, trunk, and roots (13). The software employs an image recognition algorithm in which users delineate the components of the tree (crown, trunk, and roots) directly on the digital image using a stylus or mouse. The software then automatically calculates and outputs quantitative parameters for each component, including area, height, and width, reflecting the proportional relationships between major tree parts. In psychological terms, these structural proportions correspond to self-representation, emotional stability, and perceived support—dimensions often altered in individuals experiencing depressive or suicidal states. The validity of this software has been confirmed in prior research, demonstrating high accuracy in differentiating samples compared with manual measurement, thereby supporting its clinical applicability (9).

Tree Drawing Test Suicide Ideation Assessment Scale (TDT-SIAS): Developed on the basis of prior research linking TDT features with depression and suicidal ideation (13), the TDT-SIAS (Chinese Copyright Registration No. 2023-A-000079357) includes 24 items across seven domains: overall characteristics, crown features, trunk features, root features, ground line, non-thematic features, and global features. Each item was scored based on the degree of association with suicidal ideation: presence of the indicator scored 1–5, absence scored 0, with some items graded on a 1–5 scale. A cutoff score of 9 was applied, with scores ≥9 indicating suicidal ideation.

DSM-5 (17): The *Diagnostic and Statistical Manual of Mental Disorders*, Fifth Edition (DSM-5), published by the American Psychiatric Association in 2013, was used as the diagnostic reference for depressive disorders.

M.I.N.I. suicidality module: The suicidality module of the Mini-International Neuropsychiatric Interview (M.I.N.I.) consists of six yes/no items. Respondents who answered “no” to all items were classified as “no suicide risk.” A “yes” response to any item indicated “suicide risk,” further categorized into low, moderate, or high risk. In this study, the M.I.N.I. suicidality module was used to determine the presence of suicidal ideation, and participants were accordingly classified into the “suicidal ideation group” or the “non-suicidal ideation group.”

### Statistical analysis

Data were analyzed using SPSS version 26.0. Validity indicators included sensitivity, specificity, area under the receiver operating characteristic curve (AUC), positive predictive value (PPV), and negative predictive value (NPV). The AUC and its 95% confidence interval were calculated using DeLong’s method to assess the diagnostic performance of the TDT-SIAS. Continuous variables were expressed as mean ± standard deviation (x ± s) and compared using independent-sample t-tests; categorical variables were analyzed using χ² tests. To control for potential Type I error inflation arising from multiple comparisons, Bonferroni correction was applied where appropriate. A p-value < 0.05 was considered statistically significant.

Additionally, a *post-hoc* power analysis was conducted using G*Power version 3.1 to assess the adequacy of the total sample size. Based on the observed effect size (Cohen’s d = 2.98) for the difference in total TDT-SIAS scores between groups, with α = 0.05 and total N = 104, the achieved power (1 − β) exceeded 0.99, indicating sufficient statistical power for detecting significant group differences. A two-tailed p value < 0.05 was considered statistically significant.

### Study procedure

Participants completed the tree drawing manually. Drawings were scanned and digitized. The analysis software automatically calculated quantitative indices of the crown, trunk, and roots. Trained raters then applied the TDT-SIAS to score the drawings according to standardized criteria. Finally, all data were subjected to statistical analysis.

## Results

### Demographic and clinical characteristics

A total of 50 adolescents with depressive disorders and 54 healthy controls were enrolled in this study. According to the M.I.N.I. suicidality module, 46 participants were identified as having suicidal ideation (33 males, 13 females), and 58 as having no suicidal ideation (39 males, 19 females). The two groups were comparable in terms of sex and age distribution, with no statistically significant differences between them (*p* > 0.05; **Table 1**).

**Table 1:** Baseline sample characteristics and measure scores of the participants.

Variable	Participants with suicidal ideation(n=46)	Healthy control group (n=58)	Test statistic	*p*-value
Age((years, M ± SD)	15.00(1.39)	14.80(1.43)	*t* = 0.736	0.460
Gender, n(%)			χ^2^ = 0.244	0.622
Male	33(70.0)	39(68.9)		
Female	13(30.0)	19(31.5)		

M, mean; SD, standard deviation; χ², chi-square statistic; t, t statistic.

### Validity of the tree drawing test suicide ideation assessment scale (TDT-SIAS) for screening suicidal ideation

**Figure 1:**
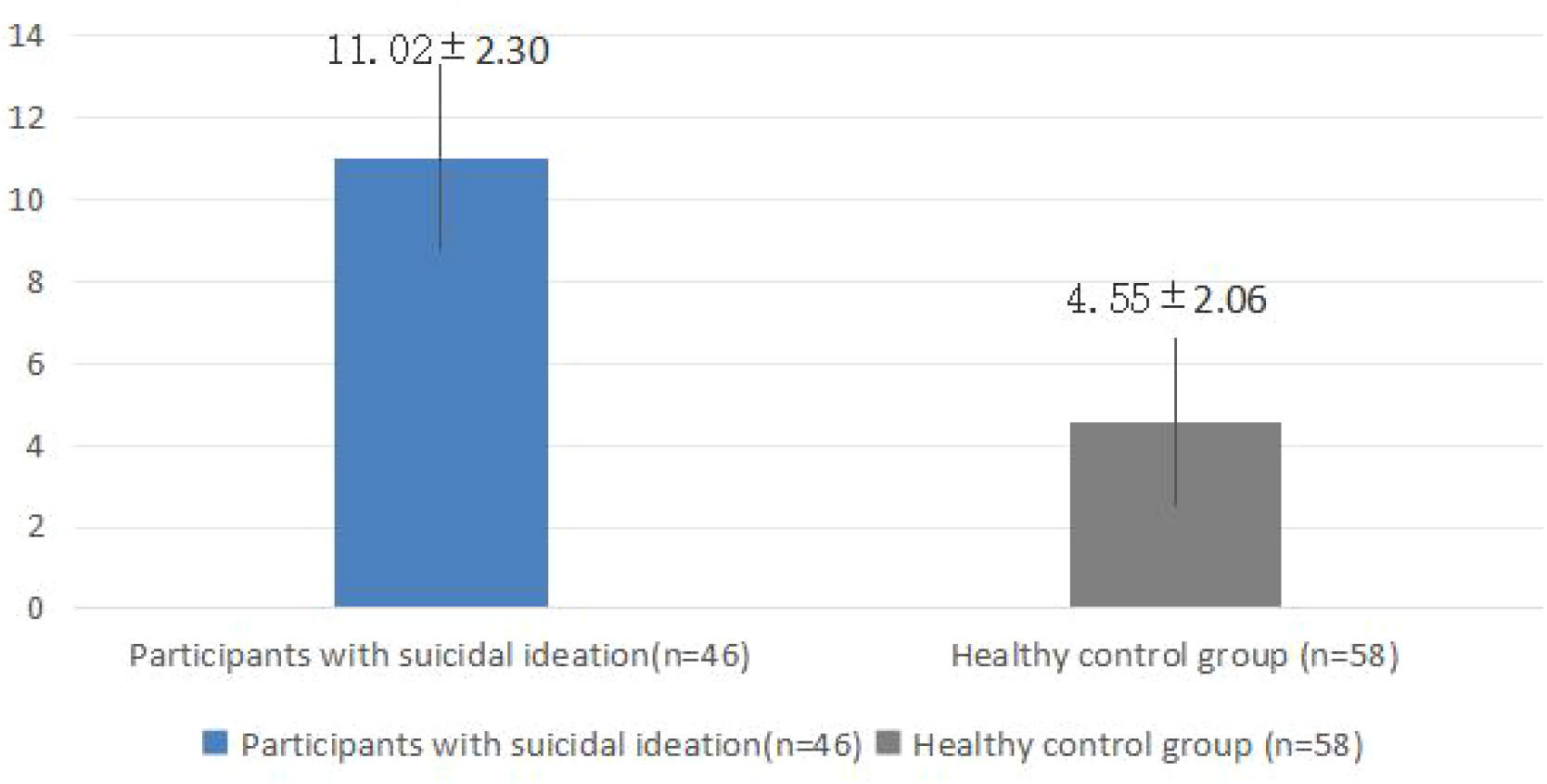
Comparison of total scores assessment between groups.

**Figure 2:**
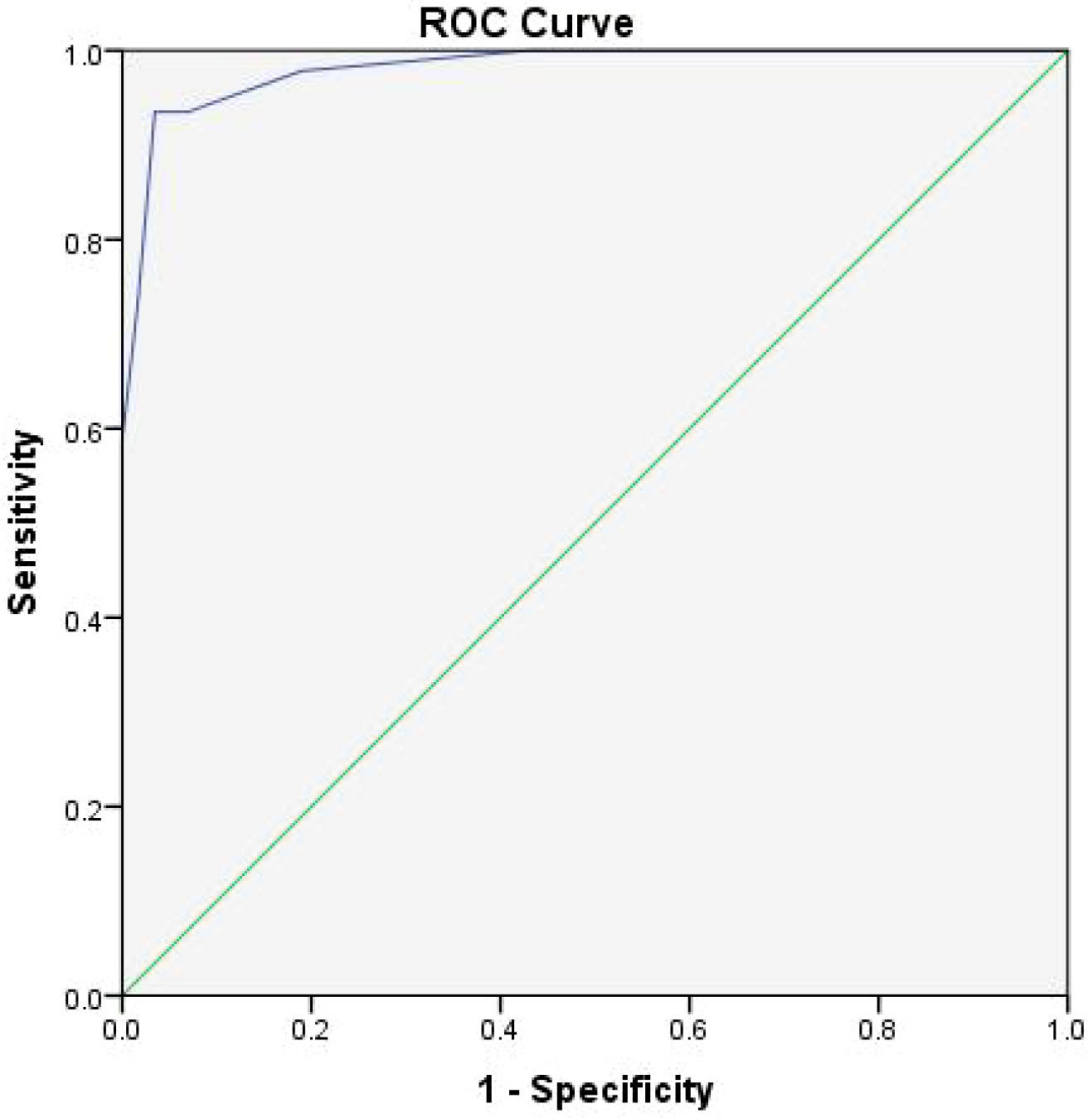
Receiver Operating Characteristic (ROC) curve for the Tree Drawing Test Suicide Ideation Assessment Scale (TDT-SIAS) in distinguishing adolescents with and without suicidal ideation.

The mean TDT-SIAS score was 11.02 ± 2.30 in the suicidal ideation group and 4.55 ± 2.06 in the non-suicidal ideation group, with a t-value of 13.364 and *p* < 0.001 (**Table 2**; **Figure 1**). Using the maximum Youden Index to determine the optimal cutoff, a score ≥ 9 (cut-off = 8.5) provided the best balance of sensitivity and specificity. At this threshold, the Youden Index was 0.901, with sensitivity of 0.935, specificity of 0.966, positive predictive value of 0.956, and negative predictive value of 0.949 (**Table 3**). The area under the ROC curve (AUC) was 0.971 (95% CI: 0.961–0.989; **Figure 2**). All reported p-values were Bonferroni-adjusted for multiple comparisons, and the significance of the results remained unchanged. For drawings with scores close to the cutoff of 9, independent raters reassessed the images to confirm the final score.

**Table 2:** Comparison of suicidal ideation assessment between groups.

Variable	Participants with suicidal ideation(n=46)	Healthy control group (n=58)	Test statistic	*p*-value
Total score(points, M ± SD)	11.02(2.30)	4.55(2.06)	*t* = 13.364	< 0.001
Assessment result, n(%)			χ^2^ = 71.063	< 0.001
Suicidal ideation(≥ 9 points)	39(84.8)	2(3.5)		
No Suicidal ideation(< 9 points)	7(15.2)	56(96.5)		

M, mean; SD, standard deviation; *χ²*, chi-square statistic; *t*, t statistic.

Total Score is based on the TDT-SIAS assessment (points).

Assessment Result: ≥9 indicates presence of suicidal ideation; <9 indicates absence.

**Table 3:** Evaluation of different cutoff points for suicidal ideation screening.

Different cutoff scores	Sensitivity	Specificity	Positive predictive value	Negative predictive value
0	1.000	0.000	0.442	
1.5	1.000	0.155	0.484	1.000
2.5	1.000	0.276	0.523	1.000
3.5	1.000	0.448	0.590	1.000
4.5	1.000	0.569	0.648	1.000
6.0	0.978	0.810	0.803	0.979
7.5	0.935	0.931	0.915	0.948
8.5	0.935	0.966	0.956	0.949
9.5	0.739	0.983	0.972	0.826
10.5	0.587	1.000	1.000	0.753
11.5	0.391	1.000	1.000	0.674
12.5	0.261	1.000	1.000	0.630
13.5	0.087	1.000	1.000	0.580
14.5	0.065	1.000	1.000	0.574
16.5	0.022	1.000	1.000	0.563
19.0	0.000	1.000		0.558

The ROC curve illustrates the diagnostic performance of the TDT-SIAS based on total scores. The area under the curve (AUC) was 0.971 (95% CI: 0.961–0.989), indicating excellent discriminative ability. The optimal cutoff score was 9, determined using the maximum Youden Index (0.901), yielding a sensitivity of 0.935 and specificity of 0.966. The diagonal reference line represents random classification (AUC = 0.5). Error bars indicate 95% confidence intervals at selected sensitivity–specificity points.

### Inter-rater reliability of the tree drawing test depression assessment scale

Prior to the initiation of the study, three graduate students majoring in psychology were recruited and underwent a one-week training session using the scoring manual, during which the scoring criteria for each item were thoroughly explained and discussed to ensure consistent understanding. Subsequently, two raters were randomly selected to independently score the tree drawings of all 104 participants. To evaluate inter-rater reliability, Cohen’s Kappa coefficients were calculated for the scores assigned by the two raters across all items. The Kappa coefficients ranged from 0.779 to 0.961,indicating good to excellent agreement between raters(see **Table 4**). As the present study primarily aimed to establish inter-rater reliability and criterion-related validity, exploratory or confirmatory factor analyses were not conducted at this stage. Given that the TDT-SIAS is a newly developed instrument with multiple drawing-based dimensions, future research with larger and more diverse samples will be conducted to examine its structural validity using EFA and CFA approaches.

**Table 4:** Rater consistency test.

Item	Kappa	*p*
1	0.926	< 0.001
2	0.938	< 0.001
3	0.917	< 0.001
4	0.907	< 0.001
5	0.899	< 0.001
6	0.887	< 0.001
7	0.961	< 0.001
8	0.955	< 0.001
9	0.892	< 0.001
10	0.948	< 0.001
11	0.924	< 0.001
12	0.951	< 0.001
13	0.849	< 0.001
14	0.903	< 0.001
15	0.911	< 0.001
16	0.907	< 0.001
17	0.779	< 0.001
18	0.783	< 0.001
19	0.843	< 0.001
20	0.901	< 0.001
21	0.922	< 0.001
22	0.807	< 0.001
23	0.891	< 0.001
24	0.954	< 0.001

## Discussion

The present study aimed to evaluate the validity of the Tree Drawing Test Suicide Ideation Assessment Scale (TDT-SIAS) for screening suicidal ideation in adolescents. A total of 46 adolescents with depressive disorders exhibiting suicidal ideation and 58 adolescents without suicidal ideation were assessed. The results demonstrated that the TDT-SIAS exhibits good validity and high consistency in identifying adolescents with suicidal ideation.

**Figure 3:**
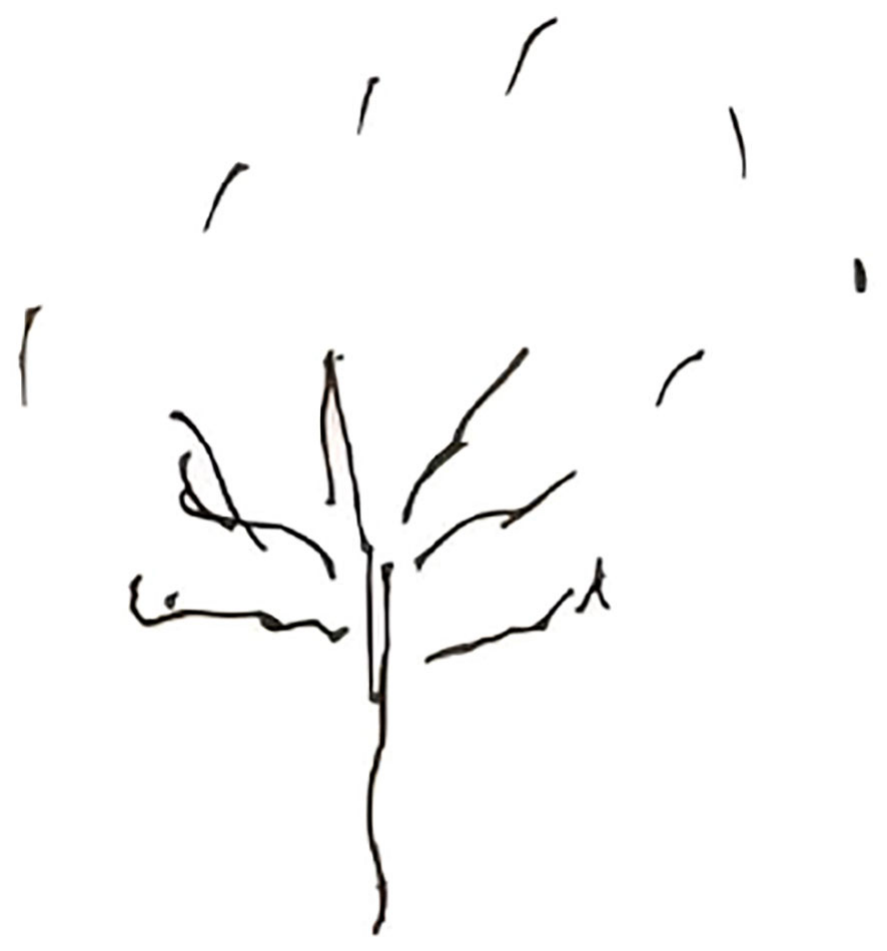
Example of a tree drawing from an adolescent with suicidal ideation (anonymized).

The TDT-SIAS is a nonverbal projective tool that captures adolescents’ self-perception, emotional state, and interpersonal orientation through tree drawings. The software-based analysis converts graphic elements—such as trunk size, root structure, and crown proportions—into quantitative indices. These structural proportions correspond to dimensions of self-representation, emotional stability, and perceived support, which are often altered in individuals experiencing depressive or suicidal states. Thus, deviations in tree structure may reflect psychological imbalance and emotional depletion, offering a window into adolescents’ inner experiences that may not be captured by traditional self-report questionnaires (**Figure 3**).

First, significant differences in total scores were observed between the suicidal ideation group and the non-suicidal ideation group (*p* < 0.001), indicating that the TDT-SIAS can effectively distinguish adolescents at risk of suicidal ideation. The mean total score of the suicidal ideation group was 11.02 ± 2.30, compared to 4.55 ± 2.06 in the non-suicidal group, reflecting a clear distinction between the two groups. Moreover, ROC curve analysis established a cutoff score of 9 to maximize both sensitivity and specificity. At this threshold, the Youden Index was 0.901, sensitivity was 0.935, specificity was 0.966, positive predictive value was 0.956, and negative predictive value was 0.949, with an area under the ROC curve (AUC) of 0.971. The AUC approaching 1 indicates excellent diagnostic accuracy, demonstrating that the TDT-SIAS performs well in both sensitivity and specificity for screening adolescent suicidal ideation (18). Notably, the high specificity (0.966) suggests that the scale can accurately identify individuals without suicidal ideation, effectively minimizing false-positive results.

In addition to its strong validity, the inter-rater reliability of the TDT-SIAS was also confirmed. Cohen’s Kappa coefficients ranged from 0.779 to 0.961, indicating high agreement between different raters. Inter-rater reliability is a critical consideration for clinical screening tools, particularly in settings where multiple evaluators may administer the assessment. Ensuring consistent scoring across raters is essential for the practical effectiveness of a screening instrument. These findings suggest that the TDT-SIAS provides reliable and stable results across different evaluators, further supporting its clinical utility as a screening tool.

Compared with existing depression and suicide screening instruments, such as the Suicidal Ideation Questionnaire (SIQ), the Beck Scale for Suicidal Ideation (BSSI), the Beck Hopelessness Scale (BHS), and the Columbia–Suicide Severity Rating Scale (C-SSRS), the TDT-SIAS demonstrated similarly high sensitivity and specificity in screening suicidal ideation among adolescents. Previous studies have shown that the SIQ and its abbreviated versions (SIQ-JR/SIQ-JR-4) exhibit high sensitivity in adolescent populations. For example, using a specific cutoff value, the SIQ-JR-4 yielded a sensitivity of 95.5% and specificity of 71.4% for predicting suicidal ideation in the past 12 months (19).In contrast, the Beck Hopelessness Scale (BHS) has been reported to show lower sensitivity and specificity than the SIQ in adolescents (20). The C-SSRS, when used in psychiatric outpatient settings, demonstrated high sensitivity and specificity (both 95%) for initial screening (21),however, its sensitivity dropped to approximately 53% during subsequent risk assessment. In psychiatric outpatient settings, item 9 of the PHQ-9, which assesses suicidal ideation, showed a sensitivity of 92% and specificity of 81%, lower than that of the C-SSRS (22).Other studies have reported that the positive predictive values of both PHQ-9 item 9 and the C-SSRS are below 25% (21).Although these instruments have its own characteristics, the high specificity and strong inter-rater reliability of the present assessment scale make it a promising and recommended screening instrument compared with currently available measures.

To provide a clearer comparison, **Table 5** summarizes the reported sensitivity and specificity of commonly used suicidal ideation screening tools in adolescents. The TDT-SIAS exhibited sensitivity (0.935) and specificity (0.966) values comparable to or higher than most established scales, indicating promising diagnostic performance within the current sample limitations. Unlike verbal self-report instruments, the TDT-SIAS offers a nonverbal, projective format that may capture implicit emotional and cognitive cues less accessible through questionnaire-based assessments. This distinction highlights the complementary value of the TDT-SIAS in multidisciplinary suicide risk screening frameworks.

**Table 5:** Sensitivity and specificity of TDT-SIAS and established suicidal ideation screening tools.

Instrument	Sensitivity	Specificity	Population/Context	Reference
TDT-SIAS (present study)	0.935	0.966	Adolescents (China)	Current study
SIQ-JR-4	0.955	0.714	Adolescents	(19)
BHS	0.65	0.72	Adolescents	(20)
C-SSRS (initial screen)	0.95	0.95	Psychiatric outpatients	(21)
C-SSRS (follow-up)	0.53	0.95	Psychiatric outpatients	(21)
PHQ-9 item 9	0.92	0.81	Psychiatric outpatients	(22)

Reported sensitivity and specificity values are derived from published studies involving adolescent or psychiatric outpatient samples. Exact estimates may vary by study design, population, and cutoff criteria. Data for SIQ-JR-4 (19), BHS (20), C-SSRS (21), and PHQ-9 item 9 (22) are presented as representative values to facilitate comparison.

As shown in **Table 3**, sensitivity and specificity varied slightly across cutoff scores ranging from 8 to 10. When the cutoff was set at 8, sensitivity was 0.935 and specificity 0.931; at a cutoff of 9, sensitivity remained 0.935 while specificity increased to 0.966; at a cutoff of 10, sensitivity decreased further to 0.739, whereas specificity rose to 0.983. These findings suggest that, although a cutoff score of 9 is statistically optimal, individuals with borderline scores may yield false-negative or false-positive results. Therefore, caution is warranted when interpreting borderline cases, and it may be advisable to combine TDT-SIAS results with other assessment methods or follow-up evaluations when necessary.

Despite the clinical relevance of these findings, several limitations should be noted. First, the relatively small sample size may limit the generalizability of the results. Second, because the case group was recruited from hospitalized adolescents with depressive disorders while controls were community-based, potential selection bias cannot be entirely ruled out despite demographic matching. In addition, all participants were recruited from a single hospital within the same region, resulting in a relatively homogeneous cultural background, which may further limit the generalizability of the findings. Future studies should consider including outpatient or non-clinical samples from multiple centers to minimize such bias and improve external validity (23, 24). Furthermore, the interpretation of tree components—such as crown size, trunk dimensions, and root structure—may be influenced by cultural norms and symbolic conventions, even when reflecting psychological constructs like self-representation, emotional stability, and perceived support. For example, adolescents from different cultural backgrounds may depict tree proportions differently due to culturally shaped drawing styles, educational experiences, or artistic conventions, which could affect the TDT-SIAS scores independently of actual psychological state. These culturally specific variations highlight the need for cautious interpretation and potential normative adjustment when applying the TDT-SIAS across diverse populations.

Additionally, potential confounding factors such as depression severity, medication status, and levels of social support may influence the association between TDT-SIAS scores and suicidal ideation. For instance, adolescents with more severe depressive symptoms or those taking psychotropic medications might produce drawings with altered structural features, independent of their suicidal ideation. Similarly, variations in perceived social support could affect tree representations, particularly the depiction of roots or crown size, which may reflect interpersonal connectedness. Future studies should systematically assess and control for these potential confounders to clarify the specific contribution of suicidal ideation to TDT-SIAS outcomes.

Future studies should include larger and more diverse samples from multiple regions, schools, and cultural backgrounds to further validate the generalizability and cultural applicability of the TDT-SIAS. Additionally, cross-cultural validation studies are warranted to examine how cultural symbolism, local ecological familiarity, and artistic conventions influence tree drawing patterns. In addition, multi-center replication studies and longitudinal follow-up designs are warranted to evaluate the predictive validity of TDT-SIAS scores for future suicidal behaviors. Integrating the TDT-SIAS with artificial intelligence–based image analysis could also enhance objectivity and efficiency in large-scale screening. Furthermore, combining the TDT-SIAS with standardized clinical interviews (e.g., the M.I.N.I. suicidality module), self-report questionnaires, and parent/teacher reports may contribute to a comprehensive, multi-method assessment framework, thereby improving both accuracy and clinical utility in suicide risk detection.

In summary, the TDT-SIAS demonstrated promising diagnostic performance within the current sample limitations in screening suicidal ideation among adolescents, exhibiting high sensitivity, specificity, and inter-rater reliability. As a non-verbal, low-cost, and easy-to-administer psychological screening tool, it offers unique advantages for adolescents, particularly for those who have difficulty expressing themselves in traditional questionnaires or who tend to conceal their emotions. Nevertheless, to ensure comprehensive and accurate assessment, the TDT-SIAS is best used as a preliminary screening tool in combination with other clinical evaluation methods. Specifically, in school-based mental health screenings and early intervention programs, integrating interview-based assessments, behavioral observations, and multi-informant reports may further enhance the sensitivity of suicidal ideation detection and improve the timeliness of interventions.

## Data Availability

The original contributions presented in the study are included in the article/supplementary material. Further inquiries can be directed to the corresponding authors.
